# Association of rs2282679 A>C polymorphism in vitamin D binding protein gene with colorectal cancer risk and survival: effect modification by dietary vitamin D intake

**DOI:** 10.1186/s12885-018-4026-1

**Published:** 2018-02-06

**Authors:** Yun Zhu, Peizhong Peter Wang, Guangju Zhai, Bharati Bapat, Sevtap Savas, Jennifer R. Woodrow, Peter T. Campbell, Yuming Li, Ning Yang, Xin Zhou, Elizabeth Dicks, John R. Mclaughlin, Patrick S. Parfrey

**Affiliations:** 1 Division of Community Health and Humanities, Faculty of Medicine, Memorial University of Newfoundland, St. John’s, NL Canada; 2 School of Public Health, Tianjin Medical University, Tianjin, China; 3 Discipline of Genetics, Faculty of Medicine, Memorial University of Newfoundland, St. John’s, NL Canada; 4 Department of Laboratory Medicine and Pathobiology, Department of Surgery, University of Toronto, Toronto, ON Canada; 5 Discipline of Oncology, Faculty of Medicine, Memorial University of Newfoundland, St. John’s, NL Canada; 6 Epidemiology Research Program, American Cancer Society, Atlanta, GA USA; 7 Tianjin Key Laboratory of Cardiovascular Remodeling and Target Organ Injury, Pingjin Hospital Heart Center, Logistics University of Chinese People’s Armed Police Force, Tianjin, China; 8 Clinical Epidemiology Unit, Faculty of Medicine, Memorial University of Newfoundland, St. John’s, NL Canada; 9 Division of Epidemiology, Public Health Ontario, Toronto, ON Canada

**Keywords:** Vitamin D binding protein, Genetic polymorphism, Colorectal cancer, Dietary vitamin D, Microsatellite instability, *BRAF* mutations

## Abstract

**Background:**

The rs2282679 A>C polymorphism in the vitamin D binding protein gene is associated with lower circulating levels of vitamin D. We investigated associations of this SNP with colorectal cancer (CRC) risk and survival and whether the associations vary by dietary vitamin D intake and tumor molecular phenotype.

**Methods:**

A population-based case-control study identified 637 incident CRC cases (including 489 participants with follow-up data on mortality end-points) and 489 matched controls. Germline DNA samples were genotyped with the Illumina Omni-Quad 1 Million chip in cases and the Affymetrix Axiom® myDesign™ Array in controls. Logistic regression examined the association between the rs2282679 polymorphism and CRC risk with inclusion of potential confounders. Kaplan-Meier curves and multivariable Cox models assessed the polymorphism relative to overall survival (OS) and disease-free survival (DFS).

**Results:**

The rs2282679 polymorphism was not associated with overall CRC risk; there was evidence, however, of effect modification by total vitamin D intake (*P*_interaction_ = 0.019). Survival analyses showed that the C allele was correlated with poor DFS (per-allele HR, 1.36; 95%CI, 1.05–1.77). The association of rs2282679 on DFS was limited to *BRAF* wild-type tumors (HR, 1.58; 95%CI, 1.12–2.23). For OS, the C allele was associated with higher all-cause mortality among patients with higher levels of dietary vitamin D (HR, 2.11; 95%CI, 1.29–3.74), calcium (HR, 1.93; 95%CI, 1.08–3.46), milk (HR, 2.36; 95%CI, 1.26–4.44), and total dairy product intakes (HR, 2.03; 95%CI, 1.11–3.72).

**Conclusion:**

The rs2282679 SNP was not associated with overall CRC risk, but may be associated with survival after cancer diagnosis. The association of this SNP on survival among CRC patients may differ according to dietary vitamin D and calcium intakes and according to tumor *BRAF* mutation status.

## Background

Colorectal cancer (CRC) is a complex, multifactorial disease resulting from multiple genetic and environmental factors [1]. Vitamin D from diet, supplements, and cutaneous synthesis from sunlight, is associated with lower risks of CRC incidence [2–5] and mortality [6–11]. The anti-carcinogenic effects of vitamin D might vary by the vitamin D-binding protein (DBP) [12]. As the major carrier protein in systemic circulation, DBP reversibly binds and transports vitamin D metabolites to different target organs, including the colorectum, thereby influencing the bioavailability of active 25-hydroxyvitamin D (25(OH)D) [12, 13]. Additionally, DBP is the precursor molecule of a potent macrophage-activating factor (GcMAF) [14], which is highly tumoricidal against various malignancies through its ability to inhibit endothelial angiogenesis [15, 16] and stimulate the inflammation-primed phagocytic activity of tumoricidal macrophages [17]. Therefore, DBP would be hypothesized to play an important role in CRC initiation and progression, either alone or in combination with vitamin D [18].

The gene encoding DBP, *GC* gene, is highly polymorphic. The single nucleotide polymorphism (SNP) rs2282679 A>C is one of the most commonly studied variants in this gene, which has been shown to be robustly correlated with serum levels of 25(OH)D in recent genome-wide association studies (GWAS) [19, 20]; specifically, the C allele of this SNP is associated with lower levels of 25(OH)D [20]. Prior studies on *GC* variants have been performed on melanoma [21], prostate [22] and breast cancers [23, 24]; however, we found only two studies that evaluated the specific association of the *GC* rs2282679 polymorphism with CRC risk, with both studies reporting no evidence of association [25, 26]. Another study reported that the *GC* rs2282679 SNP was associated with prognosis for patients diagnosed with stages II and III colon cancer [27].

Microsatellite instability (MSI) and *BRAF* V600E hotspot mutation are important molecular classifiers in CRC, which define distinct CRC subgroups arising from different oncogenic pathways. Microsatellite unstable (MSI-H) tumors are generally associated with superior prognosis [28] whereas *BRAF*-mutated cancers are related to inferior survival [29, 30]. Therefore, it is plausible that factors associated with CRC risk and survival differ across tumor molecular subtypes defined by MSI and *BRAF* mutation status. Prior studies have reported that the associations between genetic variations in vitamin D and calcium metabolic pathway and CRC vary according to MSI status, with significant associations for microsatellite unstable CRC only [31, 32]. However, no study has yet evaluated the relationship between the *GC* rs2282679 polymorphism and CRC by these tumor molecular alternations.

In this analysis, we assessed the associations of the *GC* rs2282679 variant with CRC risk and survival. We additionally evaluated the potential influence of this SNP according to dietary vitamin D, calcium, milk, and total dairy product intake and whether associations varied by tumor microsatellite instability (MSI) or *BRAF* Val600Glu mutation status.

## Methods

### Study participants

Study data and biologic specimens were drawn from the Newfoundland and Ontario Familial Colorectal Cancer Study (NFCCS), a large population-based case-control study designed to identify genetic and environmental risk and prognostic factors for CRC [33, 34]. The detailed rationale and methodology of NFCCS has been described elsewhere [4, 33, 35–37]. For the current study, only the participants from the NL portion were analyzed. Briefly, men and women with pathologically confirmed CRC were identified through the Newfoundland Familial Colorectal Cancer Registry (NFCCR). Eligibility criteria included patients newly diagnosed with CRC from 1999 to 2003 and aged 20–75 years at the time of diagnosis. Controls were selected by random digit dialing and matched on age (±5 years) and sex with cases at baseline [38]. All consenting participants were sent self-administered risk factor questionnaires (a Food Frequency Questionnaire (FFQ), a Family History Questionnaire (FHQ), and a Personal History Questionnaire (PHQ)), and were asked to provide blood samples and for permission to access their tumor specimens and medical records (for cases). A total of 656 cases and 696 controls completed detailed questionnaires and donated a blood sample. Of the 656 cases, 490 were followed for mortality and recurrence from the date of cancer diagnosis to April 2010. Vital status (i.e., death, recurrence, and metastasis) was ascertained through periodic follow-up questionnaires (e.g., FHQ), local newspapers, death certificates, pathology records, autopsy records, physicians’ notes, surgical reports, and from records at the Dr. H. Bliss Murphy Cancer Care Foundation. The main study survival outcomes were death from all-causes (i.e., overall survival (OS)) and disease-free survival (DFS), defined as death, recurrence, or metastasis (whichever came first). Follow-up time began at CRC diagnosis, and individuals who were lost to follow-up or did not die, had a recurrence or had a metastasis were censored at the time of their last contact.

Exclusions were made if patients had equivocal genotype or clinical outcome, or failed to provide sufficient information on other critical predictors. Thus, 637 cases and 489 controls for risk analyses and 489 patients for survival analysis were included in the final study.

### Diet assessment and baseline information collection

Information on diet and other lifestyle, medical and demographic characteristics was gathered with self-administered questionnaires. The dietary questionnaire was an adaptation of the Hawaii semi-quantitative FFQ to assess the dietary habits of participants from a year prior to disease diagnosis (cases) or interview (controls), which has been validated in a prior study [39]. The FFQ contained questions regarding the brand and frequency of consumption of 170 foods and beverages plus multivitamin and individual vitamin supplements [40]. The nutrient intakes from diet were calculated by multiplying the frequency of consumption of each food item by the nutrient content per average unit [4]. Total daily nutrient intakes were computed by incorporating supplement use in addition to intakes from diet. The PHQ collected information from each participant on socio-demographics (e.g., age, gender, ethnicity, and education attainment), medical conditions, bowel screening history, aspirin use, physical activity, and recent or prior alcohol and tobacco use. The FHQ gathered baseline and follow-up family history data from the participants.

### Genotyping

Genotyping for the *GC* rs2282679 allele was conducted using the Illumina Human Omni-Quad Beadchip that contains about 1.1 million SNPs at Centrillion Biosciences (USA). Control individuals were genotyped in the Laboratory of Dr. Stephen Gruber (Director, USC Norris Comprehensive Cancer Center, Los Angeles) using the Affymetrix Axiom® myDesign™ GW Array Plate, which contains 1.3 million probes. To monitor quality and consistency between the two platforms, DNA samples from 200 CRC patients were typed on both platforms. As the DNA from cases and controls were genotyped on different platforms, a genotype imputation strategy was implemented to integrate the two datasets using IMPUTE2 [41] with multi-population reference panels from 1000 Genomes (Phase 1). The imputation approach was validated based on the overlapping SNPs between the two platforms and the genotypes from 200 CRC samples that were typed on both platforms. SNPs with genotype concordance < 97% across the two platforms were removed from further analysis. For the purpose of the current study, directly measured data from both arrays on rs2282679 were retrieved from the genome-wide SNP genotype database of the NFCCR.

Our protocol for MSI and *BRAF* V600E mutation analyses in tumor DNA has been described previously [42–44]. MSI status was evaluated with 5 to 10 microsatellite markers. Tumors were deemed MSI-high if ≥30% of the repeats were unstable and MS-stable/MSI-low if < 30% of the repeats were unstable. The c.1799 T > A variant (Val600Glu mutation) region of the *BRAF* gene was amplified by *BRAF* allele-specific polymerase chain reaction technique [44].

### Statistical analysis

Group comparisons between cases and controls were performed with two-sample t test for continuous variables and Chi-square (χ^2^) test for categorical variables. The Hardy-Weinberg Equilibrium for rs2282679 genotype was evaluated using χ^2^ goodness-of-fit test. Unconditional logistic regression was used to estimate the association between the rs2282679 *GC* SNP and risk for CRC as odds ratio (OR) with 95% confidence interval (CI). Initially, logistic regression models only included genotype, age and sex. More complex models also included family history of CRC, screening procedure, multivitamin use, folic acid intake, smoking history, and education attainment. These covariates were retained in the final models because they entered the model at *P* < 0.1, altered the parameter estimates by > 10%, and/or improved the model fit.

In survival analysis, survival curves were constructed with the Kaplan–Meier method. The log-rank test and the Cox regression models were used for univariable and multivariable survival analyses to assess the association between the SNP of interest and OS and DFS of CRC. The assumption of proportional hazards for each Cox model was verified by testing the statistical significance of time-dependent covariates in the model. The hazard rate ratio (HR) and 95% CI were calculated from the Cox models. As the true inheritance mode of the rs2282679 polymorphism has not yet been established, the SNP was analyzed for risk and survival under dominant, additive, and recessive models. Given the limited sample size in some subgroups, we combined those who carried at least one of the minor C alleles in stratified analysis by selected tumor molecular phenotype. Linear trend for gene dose effect was tested by modeling ordinal variables of allele dose (0, 1, and 2) as a continuous variable. Gene-environment interactions were tested by introducing a multiplicative interaction term into the model and assessing its significance with the Wald method. Two-sided exact *P* < 0.05 was considered statistically significant. We did not adjust for multiple comparisons because the sub-tests in the current study are not independent of each other since the stratified variables are highly correlated (i.e., vitamin D, calcium, and dairy products). Although adjustment for multiple testing reduces type I error, it increases type II error and errors of interpretation [45]. All data management and analyses were performed using SAS software, Version 9.3.

## Results

The rs2282679 polymorphism was in Hardy-Weinberg equilibrium (*P* > 0.05). Among controls, the genotype frequency was 7.4% homozygous (CC), 42.5% heterozygous (AC) and 50.1% wild-type homozygous (AA); the observed minor allele frequencies in the controls were comparable to that previously reported [25]. During a maximum follow-up of 10.9 years (mean: 6.3 years), 150 deaths occurred among the 489 patients included in the survival analysis. The cause of death defined by ICD codes was obtained for 105 of 150 deceased patients; thereof the majority (90.5%) was due to CRC.

### Baseline characteristics of cases and controls

Cases and controls had similar sex and ethnicity distributions, and the majority reported their race as White (Table 1). Relative to cases, controls were slightly younger, leaner (lower body mass index), better educated, less likely to smoke, and more likely to have had a colorectal cancer screening/early detection procedure. Among those who completed the FFQ, total vitamin D and calcium intakes were significantly higher in controls than in cases (*P* = 0.001). Family history of CRC (first-degree relatives affected only) was reported by 9.8% of the patients and 7.5% of the controls. MSI-high was identified in 61 of 507 (12.0%) tumors, and *BRAF* Val600Glu mutation was detected in 10.8% of tumors.

**Table 1:** Selected demographical and clinicopathological characteristics of study population (cases and controls)

Characteristics	Cases	Controls	*P*-value^a^
	No.(%)	No.(%)	
Age (year)^b^	63.1 ± 8.6	61.2 ± 9.0	0.001
BMI (kg/m^2^)^b^	28.1 ± 5.0	27.3 ± 4.5	0.003
Sex			
Men	399 (62.9)	272 (58.5)	
Women	235 (37.1)	193 (41.5)	0.136
Race			
White	615 (97.0)	444 (95.5)	
Other	19 (3.0)	21 (5.0)	0.184
Family history of CRC			
Yes	62 (9.8)	35 (7.5)	
No	572 (90.2)	430 (92.5)	0.194
Level of education			
Lower than high school	302 (47.8)	140 (30.2)	
High-school graduate	100 (15.8)	74 (16.0)	
College	177 (28.0)	175 (37.7)	
Bachelor or higher	53 (8.4)	75 (16.1)	< 0.001
Smoking history			
Current	129 (20.3)	58 (12.5)	
Former	325 (51.3)	228 (49.4)	
Never	180 (28.4)	176 (38.1)	< 0.001
Reported screening procedure			
Yes	66 (10.4)	107 (23.0)	
No	568 (89.6)	358 (77.0)	< 0.001
Fruit intake (servings ^c^/wk)^b^	9.7 ± 8.3	10.9 ± 8.1	0.027
Total vitamin D intake (μg/d)^b^	8.7 ± 6.4	10.2 ± 7.9	0.001
Total calcium intake (mg/d)^b^	1019.8 ± 488.8	1097.1 ± 567.7	0.018
Milk (g/d)^b^	287.1 ± 278.0	299.9 ± 295.5	0.465
Total dairy products (g/d)^b^	364.5 ± 303.3	389.9 ± 340.0	0.200
Tumor location			
Colon	417 (65.8)	–	–
Rectum	217 (34.2)	–	–
Stage at diagnosis			
I/II	288 (58.9)	–	–
III/IV	201 (41.1)	–	–
Chemoradiotherapy			
Yes	106 (19.9)	–	–
No	427 (80.1)	–	–
MSI status			
MSS/MSI-L	446 (88.0)	–	–
MSI-H	61 (12.0)	–	–
*BRAF* mutation status			
Wild type	445 (89.2)	–	–
*BRAF* mutant	54 (10.8)	–	–

*Abbreviations*: *BMI* body mass index, *CRC* colorectal cancer, *MSI* microsatellite instability, *MSI-H* microsatellite instability-high, M*SS/MSI-L* microsatellite stable/microsatellite instability-low

^a^*P*values are for the significance of the two-sample t test for continuous variables and of the chi-square test for categorical variables

^b^Continuous variables presented as mean ± s.d. (standard deviation)

^c^A serving of fruit is: 1 medium-sized fresh fruit; ½ cup of chopped, cooked or canned fruit; ¼ cup of dried fruit; 6 oz of fruit juice (50%–100% pure juice)

Subjects with missing values are not reported in the table

### Association of rs2282679 genotype with CRC risk

The rs2282679 SNP was not associated with risk of CRC overall or when stratified by MSI or *BRAF*-mutation subtypes (Table 2). Specifically, the odds ratio was 1.10 (95% CI, 0.88–1.37) per variant C allele and 1.21 (95% CI, 0.70–2.09) in CC homozygotes compared with AA homozygotes. The ORs were similar for men and women and did not differ according to tumor anatomical sub-site (data available upon request).

**Table 2:** Frequency distribution and associations of *GC* SNP rs2282679 with CRC risk (overall and by molecularly defined subtypes)

rs2282679 Genotype/Allele	Cases N(%)	Controls N(%)	OR (95% CI)^a^	OR (95% CI)^b^
All cases vs. controls				
AA	309 (48.5)	245 (50.1)	1.00	1.00
AC	282 (44.3)	208 (42.5)	1.08 (0.82–1.41)	1.10 (0.83–1.47)
CC	46 (7.2)	36 (7.4)	1.06 (0.63–1.77)	1.21 (0.70–2.09)
*P*_trend_^c^			0.647	0.403
CC + AC (vs. AA)	328 (51.5)	244 (49.9)	1.07 (0.83–1.39)	1.12 (0.85–1.47)
CC (vs. AC + AA)	46 (7.2)	36 (7.4)	1.02 (0.62–1.68)	1.15 (0.68–1.97)
Per C allele			1.05 (0.85–1.29)	1.10 (0.88–1.37)
*BRAF* V600E mutant cases vs. controls				
AA	25 (46.3)	245 (50.1)	1.00	1.00
CC + AC^d^	29 (53.7)	244 (49.9)	1.03 (0.54–1.96)	1.10 (0.56–2.16)
*BRAF* wild-type cases vs. controls				
AA	223 (50.1)	245 (50.1)	1.00	1.00
CC + AC^d^	222 (49.9)	244 (49.9)	1.08 (0.82–1.42)	1.10 (0.82–1.47)
MSI-H cases vs. controls				
AA	31 (50.8)	245 (50.1)	1.00	1.00
CC + AC^d^	30 (49.2)	244 (49.9)	0.99 (0.56–1.80)	1.03 (0.56–1.90)
MSS/MSI-Low cases vs. controls				
AA	223 (50.0)	245 (50.1)	1.00	1.00
CC + AC^d^	223 (50.0)	244 (49.9)	1.09 (0.83–1.44)	1.15 (0.86–1.53)

*Abbreviations*: *CRC* colorectal cancer, *OR* odds ratio, *MSI-H* microsatellite instability-high, *MSS/MSI-L* microsatellite stable/microsatellite instability-low

^a^Crude model adjusted for age and sex

^b^Multivariable model additionally adjusted for family history of CRC, screening procedure, multivitamin use, folic acid intake, smoking history, and education attainment where applicable

^c^Linear trend tested by modeling the ordinal variables of genotype dose as a continuous variable

^d^CC and AC genotypes were analyzed jointly because of limited sample size in some subgroups

### Interactions of rs2282679 genotype with dietary characteristics in relation to CRC risk

A previous NFCCS study demonstrated that total vitamin D intake was inversely associated with CRC incidence [4]. We therefore cross classified subjects on total vitamin D intake and other related dietary factors with rs2282679 genotype for CRC risk (Table 3). Among participants with the more common AA genotype, we confirmed the association, observing a 2.5-fold increased risk of CRC in individuals consuming total vitamin D in the lowest tertile than in those in the highest tertile (95% CI, 1.52–4.09). Among carriers of the C allele, however, no appreciable difference in ORs between strata of total vitamin D intake was observed. Intriguingly, carrying the risk allele (i.e. the heterozygous/homozygous genotypes) conferred an enhanced risk for this malignancy in the presence of high level of vitamin D intake (OR, 1.65; 95% CI, 1.01–2.71; *P*_interaction_ = 0.019). Stratification on consumption of milk and total dairy products suggested borderline significant effect modifications (*P*_interaction_ = 0.056 for milk and 0.079 for total dairy products). Additionally, there was no evidence that the associations of this SNP with CRC risk were modified by body mass index (obese vs. not obese), drinking status (non-drinkers vs. drinkers) or smoking history (non-smokers vs. smokers) (data available upon request).

**Table 3:** Risk estimates for interactions between *GC* rs2282679 genotypes and non-genetic dietary factors

	*GC* rs2282679 genotype		*P*_interaction_^b^
	AA OR (95% CI)^a^	AC + CC OR (95% CI)^a^	
Total vitamin D intake (μg/d)			
Highest tertile (≥10.1)	1.00	**1.65 (1.01–2.71)**	
Lowest tertile (< 5.0)	**2.50 (1.52–4.09)**	**1.72 (1.03–2.90)**	0.019
Total calcium intake (mg/d)			
Highest tertile (≥1195.4)	1.00	1.45 (0.89–2.36)	
Lowest tertile (< 761.4)	1.21 (0.73–2.00)	1.63 (0.95–2.81)	0.827
Milk (g/d)			
Highest tertile (≥321.3)	1.00	1.61 (0.99–2.61)	
Lowest tertile (< 146.5)	1.46 (0.90–2.38)	1.21 (0.72–2.04)	0.056
Total dairy products (g/d)			
Highest tertile (≥432.0)	1.00	1.61 (0.99–2.61)	
Lowest tertile (< 210.6)	1.44 (0.90–2.32)	1.27 (0.75–2.14)	0.079

*Abbreviations*: *CRC* colorectal cancer, *OR* odds ratio

^a^Adjusted for age, sex, family history of CRC, screening procedure, folic acid intake, smoking history, and education attainment where applicable

^b^*P* for interaction is computed with Wald method testing significance of multiplicative interaction term between *GC* SNP rs2282679 genotype and respective stratified variable

Those with *P* < 0.05 are in bold

### Association of rs2282679 genotype and survival outcome

**Fig. 1:**
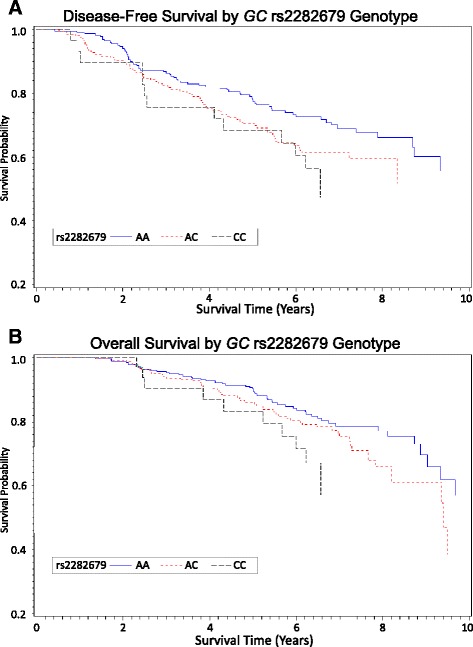
Survival curves for (**a**) disease-free survival and (**b**) overall survival by *GC* rs2282679 genotypes. Adjusted for sex, age at diagnosis, tumor stage at diagnosis, marital status, race, reported chemoradiotherapy, MSI status, *BRAF* mutation status, tumor location, fruit intake, and body mass index

**Fig. 2:**
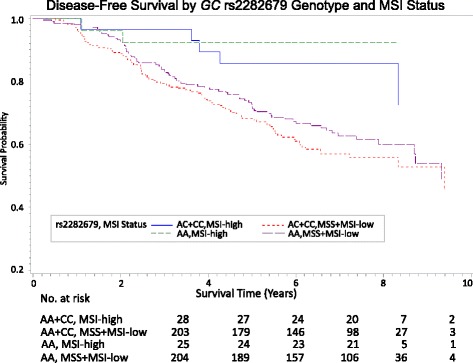
Kaplan-Meier survival curves for disease-free survival according to *GC* rs2282679 genotypes and MSI status

Survival analysis showed a positive association between rs2282679 polymorphism and reduced DFS of CRC, with the co-dominant CC and the dominant CC + AC vs. AA model exhibiting 1.93- and 1.40- fold increases in the risk for DFS, respectively (Table 4, Fig. 1). The per-allele HR was 1.36 (95% CI, 1.05–1.77; *P*_trend_ = 0.020). The adverse prognosis in relation to the risk allele was limited to *BRAF* wild-type tumors (HR, 1.58; 95% CI, 1.12–2.23). Among patients harboring the *BRAF* Val600Glu mutation, the DFS was essentially the same for individuals with and without the C allele (HR, 0.95; 95% CI, 0.25–3.62; *P*_interaction_ = 0.043). Although no evidence existed for a differential prognostic role of rs2282679 according to MSI status, patients with the AA genotype/MSI-high tumors experienced the most favorable DFS whereas patients with AC + CC genotypes/MSS + MSI-low tumors had the worst DFS (Fig. 2). The 5-year DFS of CRC was 92% for AA/MSI-high, 82% for AC + CC genotypes/MSI-high, 68% for AA genotype/MSS + MSI-low, and 65% for AC + CC genotypes/MSS + MSI-low tumors (Log-rank *P* = 0.0013, Fig. 2). Our results did not confirm a prognostic relevance of rs2282679 in OS of CRC.

**Table 4:** Association between *GC* SNP rs2282679 genotypes and overall and disease free colorectal cancer survival (overall and stratified by tumor molecular phenotype)

rs2282679 Genotype/Allele	Disease-Free Survival		*P*_interaction_^c^	Overall Survival		*P*_interaction_^c^
	No. of Events^a^ /At Risk	HR (95% CI)^b^		No. of Events^a^ /At Risk	HR (95% CI)^b^	
Total colorectal cancer						
AA	83/245	1.00		69/245	1.00	
AC	80/207	1.33 (0.94–1.88)		68/208	1.24 (0.85–1.81)	
CC	16/36	**1.93 (1.06–3.52)**		13/36	1.60 (0.85–3.02)	
*P*_trend_^d^		0.020			0.107	
CC + AC (vs. AA)	96/243	**1.40 (1.00–1.95)**		81/244	1.29 (0.90–1.85)	
CC (vs. AC + AA)	16/36	1.69 (0.95–2.99)		13/36	1.44 (0.79–2.65)	
Per C allele		**1.36 (1.05–1.77)**			1.26 (0.95–1.66)	
*BRAF* Val600Glu mutant						
AA	8/20	1.00		5/20	1.00	
CC + AC^e^	8/26	0.95 (0.25–3.62)		7/26	1.53 (0.47–4.95)	
*BRAF* wild-type						
AA	65/200	1.00		55/200	1.00	
CC + AC ^e^	83/195	**1.58 (1.12–2.23)**	0.043	70/196	1.33 (0.93–1.91)	0.892
MSI-H						
AA	3/25	1.00		0/26	1.00	
CC + AC^e^	5/28	1.26 (0.18–8.96)		3/28	NC^f^	
MSS/MSI-L						
AA	76/204	1.00		65/204	1.00	
CC + AC^e^	87/203	1.34 (0.97–1.87)	0.702	74/204	1.26 (0.89–1.77)	0.210

*Abbreviations*: *HR* hazard rate ratios, *CI* confidence interval, *MSI-H* microsatellite instability-high, *MSS/MSI-L* microsatellite stable/microsatellite instability-low

^a^Events are defined as deaths for overall survival and death, recurrence, or metastasis (whichever occurred earliest) for disease-free survival

^b^Multivariable Cox model adjusted for sex, age at diagnosis, tumor stage at diagnosis, marital status, race, reported chemoradiotherapy, MSI status, BRAF mutation status, tumor location, fruit intake, and body mass index where applicable

^c^*P* for interaction is computed with Wald method testing significance of multiplicative interaction term between *GC* SNP rs2282679 genotype and molecular subtype

^d^Linear trend tested by modeling the ordinal variables of genotype dose as a continuous variable

^e^CC and AC genotypes were analyzed jointly because of limited sample size in some subgroups

^f^NC: not calculated

Those with *P* < 0.05 are in bold

### Interactions of rs2282679 genotype with dietary characteristics in relation to CRC survival

The *GC* rs2282679 genotype interacted with dietary factors to influence OS after CRC diagnosis (Table 5). Specifically, the positive association between carriage of the C allele and poor OS seemed limited to patients in higher categories of dietary vitamin D, calcium, milk, and total dairy product intakes; the HRs associated with the AC + CC genotypes were 2.11 (95% CI, 1.29–3.74; *P*_interaction_ = 0.040), 1.93 (95% CI, 1.08–3.46; *P*_interaction_ = 0.043), 2.36 (95% CI, 1.26–4.44; *P*_interaction_ = 0.004), and 2.03 (95% CI, 1.11–3.72; *P*_interaction_ = 0.024), respectively. Low intake of pre-diagnostic milk was associated with worse OS among wild-type homozygotes (AA) only, with the HR equaled to 2.09 (95% CI, 1.09–4.02). For DFS, effect-modification analyses yielded comparable but nonsignificant results. Analyses using total vitamin D or total calcium intakes (diet+supplements) did not show any associations (patterns) more significant or different. Therefore, we only present results stratified by dietary intakes. Stratification on tumor subsite (colon vs. rectum), reported chemoradiation therapy (yes vs. no) or smoking history (non-smokers vs. smokers) detected no significant interaction (data available upon request).

**Table 5:** Association between *GC* rs2282679 genotypes and overall and disease free colorectal cancer survival stratified by non-genetic dietary factors

	*GC* rs2282679 genotype		*P*_interaction_^b^
	AA HR (95% CI)^a^	AC + CC HR (95% CI)^a^	
Disease-Free Survival			
Dietary vitamin D (μg/d)			
Highest tertile (≥7.1)	1.00	1.65 (0.97–2.82)	
Lowest tertile (< 4.6)	1.03 (0.58–1.83)	1.08 (0.56–2.08)	0.191
Dietary calcium (mg/d)			
Highest tertile (≥1120.2)	1.00	1.58 (0.92–2.71)	
Lowest tertile (< 702.1)	1.06 (0.59–1.91)	1.14 (0.62–2.11)	0.355
Milk (g/d)			
Highest tertile (≥274.6)	1.00	1.63 (0.92–1.84)	
Lowest tertile (< 128.1)	1.21 (0.68–2.16)	0.97 (0.51–1.84)	0.083
Total dairy products (g/d)			
Highest tertile (≥411.8)	1.00	1.51 (0.87–2.60)	
Lowest tertile (< 199.2)	1.00 (0.57–1.77)	0.99 (0.53–1.83)	0.289
Overall Survival			
Dietary vitamin D (μg/d)			
Highest tertile (≥7.1)	1.00	**2.11 (1.29–3.74)**	
Lowest tertile (< 4.6)	1.28 (0.68–2.42)	1.11 (0.54–2.27)	0.040
Dietary calcium (mg/d)			
Highest tertile (≥1120.2)	1.00	**1.93 (1.08–3.46)**	
Lowest tertile (< 702.1)	1.55 (0.82–2.91)	1.18 (0.59–2.35)	0.043
Milk (g/d)			
Highest tertile (≥274.6)	1.00	**2.36 (1.26–4.44)**	
Lowest tertile (< 128.1)	**2.09 (1.09–4.02)**	1.26 (0.61–2.62)	0.004
Total dairy products (g/d)			
Highest tertile (≥411.8)	1.00	**2.03 (1.11–3.72)**	
Lowest tertile (< 199.2)	1.77 (0.94–3.33)	1.27 (0.63–2.58)	0.024

*Abbreviations*: *HR* hazard rate ratios, *CI* confidence interval

^a^Adjusted for sex, age at diagnosis, stage at diagnosis, marital status, race, reported chemoradiotherapy, MSI status, *BRAF* mutation status, tumor location, body mass index where applicable

^b^*P* for interaction is computed with Wald method testing significance of multiplicative interaction term between GC SNP rs2282679 genotype and respective stratified variable

Those with *P* < 0.05 are in bold

## Discussion

In this study, we observed no clear association of rs2282679 SNP with overall CRC risk, but noted a suggestive association of the CC genotype with DFS. A previous multicenter case-control study of 10,061 CRC cases and 12,768 controls of European ancestry found no evidence for associations between *GC* rs2282679 and the risk of CRC overall or for colon or rectal tumor separately [26]. In a Mendelian Randomization study in Scotland, Theodoratou et al. [25] reported a nonsignificant association of rs2282679 wild-type A allele with CRC risk (Per A allele: OR, 0.97; 95% CI, 0.90–1.06). The only available previous study investigating the prognostic effect of the SNP on CRC reported consistent results that *GC* rs2282679 polymorphism was significantly associated with reduced time to recurrence (HR, 3.30; 95% CI, 1.09–9.97, *P* = 0.034) in stages II and III colon cancer patients treated with surgery alone [27].

Referring to two recent GWAS studies [19, 20], the *GC* rs2282679 has been identified as the strongest genomic predictor of serum vitamin D level (*P* = 2.0 × 10^− 30^). Per copy of the risk C allele was associated with an approximately 50% elevated risk for hypovitaminosis among Caucasians [20]. In another study by Zhang et al. [46], the C allele of this SNP was associated with lower circulating DBP concentrations and thus lower 25(OH)D bioavailability to target organs. Together with the fact that vitamin D has been shown to reduce the growth of CRC xenografts by influencing cell growth, differentiation, apoptosis, as well as immune-modulation, the elevated risk of C allele carriers may be attributed to their low circulating DBP and 25(OH)D concentrations relative to noncarriers [47–49]. Alternatively, DBP can be converted to GcMAF, an activator of macrophages, by stepwise incubation of β-galactosidase and sialidase [17]. GcMAF could activate phagocytosis of macrophages during inflammation, reduce tumor growth and stimulate cell apoptosis [15, 16]. In addition, GcMAF has been demonstrated to have the potential utility as an antitumorigenic drug for metastatic breast cancer [50]. Therefore, genetic variation in *GC* may alternatively influence cancer outcome via GcMAF, a biological mechanism independent of vitamin D levels.

We found that carriage of the risk C allele was associated with an increased likelihood of CRC incidence in patients with high vitamin D intake (*P*_interaction_ = 0.019). No prior studies were found that specifically examined the interaction between rs2282679 polymorphism and vitamin D on CRC; yet, two other *GC* SNPs, rs17467825 and rs7041, which are in strong linkage disequilibrium with *GC* rs2282679 (γ^2^ = 1.0 and 0.6 respectively) [20], have been associated with a slightly greater risk of CRC among individuals who consumed total vitamin D above the median in a multicenter case–unaffected sibling control study, though the interactions were not significant [32]. In addition, low vitamin D intake conferred higher risk of CRC among wild-type AA carriers but less obvious effect among AC or CC carriers; therefore, subjects with the AC/CC genotype might derive little benefit from high vitamin D intake, which may be due to their low affinity and abundance of DBP that might influence the function of vitamin D. Previous research [51] found that serum 25(OH)D level had greater effect on colorectal adenoma among patients with high total calcium intake; it is therefore unsurprising that we also observed a particularly strong association between the variation and all-cause mortality in patients at the higher calcium category. Based on these observations, we may speculate that the influence of rs2282679 polymorphism on either carcinogenesis or progression of CRC was strengthened by a metabolically permissive environmental condition characterized by high levels of dietary vitamin D, calcium, or foods rich in vitamin D and calcium [52].

In this study, the associations between rs2282679 SNP and DFS and OS were of similar patterns but stronger with DFS than OS. The difference in results may be explained by several reasons. It is plausible that many deaths among CRC patients are preceded by tumor metastasis or recurrence. Thus, the DFS end point may be dominated by metastasis and recurrence rather than deaths from all causes [53]; the difference in outcomes may have affected the results. A second possible explanation is that the power to detect an association for OS is less than that for DFS as the OS end point requires extended follow-up [53]. Therefore, the non-significant *P* values for OS might reflect inadequate power rather than a true lack of effect.

Our data suggest that the *GC* rs2282679 variation may be associated with poor DFS among patients with *BRAF* wild-type tumors, but not among *BRAF* mutant tumors (*P*_interaction_ = 0.043). Although intriguing, the interaction of the SNP with tumor *BRAF* mutation status should be interpreted with caution because of a limited statistical power caused by low number of patients with *BRAF* mutant tumors, as well as the lack (at least to date) of exact mechanism of action underlying the prognostic value of this gene only in *BRAF* mutated CRC. Additionally, we observed an additive effect of the rs2282679 genotype combined with MSI status; unsurprisingly, the most favorable prognosis as determined by DFS was seen among patients with AA/MSI-high tumors (vs. AC + CC/MSS + MSI-low). MSI has been established as a prognostic biomarker that confers survival advantage to CRC due to increased apoptosis rate and high lymphocytic infiltration [54–57]. Our observations suggest that the prediction model of CRC outcome should additionally integrate the rs2282679 genotype. These results may provide relevant information for identification of patients with increased susceptibility to CRC incidence and mortality and for patient assignment to interventions that are tailored to the individual. Additional studies should be addressed to investigate the role of rs2282679/MSI classification in predicting the response to therapeutic lifestyle interventions.

One limitation of our study is that only one genetic variant of the *GC* gene was evaluated, thereby providing incomplete coverage of this gene; and we cannot exclude that genetic polymorphisms in other genes in the vitamin D metabolism pathway (e.g., vitamin D receptor) may also influence overall CRC initiation and progression. It is also possible that rs2282679 is not the true causal variant in itself but acts as a proxy through linkage disequilibrium (LD). Moreover, plasma 25(OH)D levels were not measured in this study. The lack of 25(OH)D measurements impeded us to test the relations of *GC* rs2282679 polymorphisms with plasma vitamin D concentration and to evaluate the extent to which the high risk of CRC mortality associated with the C allele is mediated through low 25(OH)D levels. Furthermore, dietary vitamin D intake may not accurately reflect each participant’s vitamin D status since dietary history as measured by the FFQ is imprecise, and neither dermatic synthesis of vitamin D from sun exposure nor long-term dietary vitamin D intake was taken into account. Additionally, individuals were asked to report dietary exposures from one year prior to diagnosis for cases and one year prior to recruitment for controls; therefore, cases recalled dietary intakes from years earlier than controls. The longer recall period increases the rate of recall error resulting in higher likelihood of exposure misclassification in the case group.

Among the strengths of the study is the careful data collection, with a combination of results from genotyping and epidemiologic questionnaires. The availability of information on known environmental and genetic risk factors of CRC allowed us to investigate potential gene-gene or gene-environment interactions. The relatively large sample size with up to 10 years of follow-up permitted enough power to discern the significant gene-gene and gene-environment interactions in modifying CRC risk and survival using stratified analyses, which could be missed in smaller investigations. Finally, we were able to link the *GC* rs2282679 genotype to both risk and survival of CRC to recapitulate the entire spectrum of the disease from initiation through progression [58].

## Conclusions

Our data demonstrate that the *GC* rs2282679 polymorphism is not associated with CRC risk overall, but suggest a possible reduced DFS after CRC diagnosis. These results identified an association between the *GC* SNP rs2282679 and DFS of CRC and effect modifications by vitamin D intake and *BRAF* mutation status. The genotype at the *CG* rs2282679 locus, along with vitamin D and *BRAF* mutation status, has potential utility as a susceptibility and prognostic biomarker of CRC. Future studies should verify these findings in other populations as well as clarify the molecular mechanisms behind the differential effects of the SNP on CRC outcomes according to vitamin D and *BRAF* mutation status.

## Abbreviations

CI: Confidence Interval
CRC: Colorectal Cancer
DBP: Vitamin D-Binding Protein
DFS: Disease-Free Survival
FFQ: Food Frequency Questionnaire
FHQ: Family History Questionnaire
GcMAF: Macrophage-Activating Factor
GWAS: Genome-Wide Association Study
HR: Hazard Ratio
MSI: Microsatellite Instability
NFCCR: Newfoundland Familial Colorectal Cancer Registry
NFCCS: Newfoundland and Ontario Familial Colorectal Cancer Study
OR: Odds Ratio
OS: Overall Survival
PHQ: Personal History Questionnaire
SNP: Single Nucleotide Polymorphism
